# 1-[3-(2,4-Dichloro-5-fluoro­phen­yl)-5-(3-methyl-2-thien­yl)-4,5-dihydro-1*H*-pyrazol-1-yl]ethanone

**DOI:** 10.1107/S1600536808033837

**Published:** 2008-10-22

**Authors:** N. Anuradha, A. Thiruvalluvar, M. Mahalinga, R. J. Butcher

**Affiliations:** a PG Research Department of Physics, Rajah Serfoji Government College (Autonomous), Thanjavur 613 005, Tamil Nadu, India; b SeQuent Scientific Limited, 120 A&B Industrial Area, Baikampady, New Mangalore 575 011, India; c Department of Chemistry, Howard University, 525 College Street NW, Washington, DC 20059, USA

## Abstract

In the title mol­ecule, C_16_H_13_Cl_2_FN_2_OS, the dihedral angle between the thio­phene and benzene rings is 80.34 (12)°. The pyrazoline ring is in an envelope conformation, and the plane through the four coplanar atoms makes dihedral angles of 85.13 (9) and 6.89 (10)° with the thio­phene and benzene rings, respectively. The C and O atoms of the acetyl group are nearly coplanar with the attached pyrazoline ring. In the crystal structure, inversion dimers arise from pairs of inter­molecular C—H⋯O hydrogen bonds. A short inter­molecular Cl⋯S contact of 3.4250 (13) Å is also found.

## Related literature

For a related crystal structure, see: Thiruvalluvar *et al.* (2007 ▶).
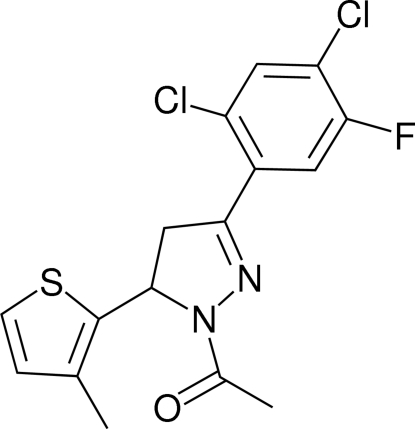


## Experimental

### 

#### Crystal data

C_16_H_13_Cl_2_FN_2_OS
*M* *_r_* = 371.25
Triclinic, 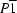

*a* = 7.2240 (5) Å
*b* = 8.8642 (4) Å
*c* = 14.0518 (9) Å
α = 100.794 (5)°
β = 103.307 (6)°
γ = 101.003 (5)°
*V* = 833.99 (10) Å^3^
*Z* = 2
Mo *K*α radiation
μ = 0.53 mm^−1^
*T* = 295 (2) K
0.52 × 0.43 × 0.35 mm

#### Data collection

Oxford Diffraction R Gemini diffractometer
Absorption correction: multi-scan (*CrysAlis RED*; Oxford Diffraction, 2008 ▶) *T*_min_ = 0.786, *T*_max_ = 1.000 (expected range = 0.654–0.831)
12358 measured reflections
5445 independent reflections
3028 reflections with *I* > 2σ(*I*)
*R*_int_ = 0.020

#### Refinement

*R*[*F* ^2^ > 2σ(*F* ^2^)] = 0.051
*wR*(*F* ^2^) = 0.179
*S* = 1.12
5445 reflections
210 parameters
H-atom parameters constrained
Δρ_max_ = 0.32 e Å^−3^
Δρ_min_ = −0.39 e Å^−3^

### 

Data collection: *CrysAlis CCD* (Oxford Diffraction, 2008 ▶); cell refinement: *CrysAlis RED* (Oxford Diffraction, 2008 ▶); data reduction: *CrysAlis RED*; program(s) used to solve structure: *SHELXS97* (Sheldrick, 2008 ▶); program(s) used to refine structure: *SHELXL97* (Sheldrick, 2008 ▶); molecular graphics: *ORTEP-3* (Farrugia, 1997 ▶); software used to prepare material for publication: *PLATON* (Spek, 2003 ▶).

## Supplementary Material

Crystal structure: contains datablocks global, I. DOI: 10.1107/S1600536808033837/wn2286sup1.cif

Structure factors: contains datablocks I. DOI: 10.1107/S1600536808033837/wn2286Isup2.hkl

Additional supplementary materials:  crystallographic information; 3D view; checkCIF report

## Figures and Tables

**Table 1:** Hydrogen-bond geometry (Å, °)

*D*—H⋯*A*	*D*—H	H⋯*A*	*D*⋯*A*	*D*—H⋯*A*
C2—H2*A*⋯O1^i^	0.96	2.58	3.533 (4)	171

Symmetry code: (i) 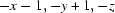.

**Fig. 1.**
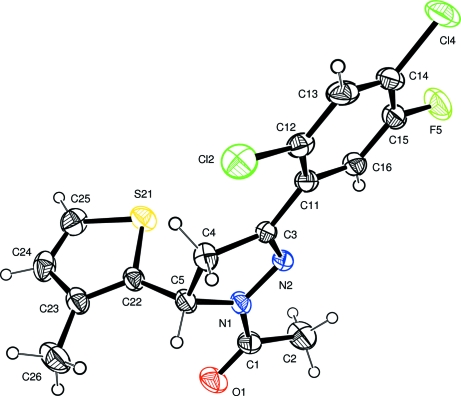
The molecular structure of the title compound, showing the atom-numbering scheme and displacement ellipsoids drawn at the 30% probability level. H atoms are shown as small spheres of arbitrary radius.

**Fig. 2.**
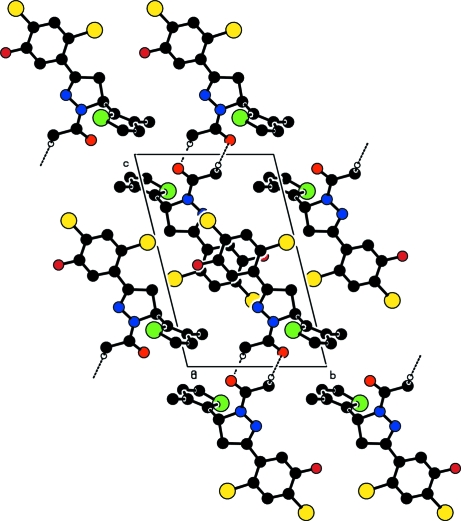
The packing of the title compound, viewed down the a axis. Dashed lines indicate hydrogen bonds. H atoms not involved in hydrogen bonding have been omitted.

## supplementary crystallographic information

### Comment

A great deal of attention has been paid to the synthesis and structural aspects of pyrazolines, as witnessed by continued activity in this area (Thiruvalluvar *et al.*, 2007).

In the title molecule, C_16_H_13_Cl_2_FN_2_OS, Fig.1., the dihedral angle between the thiophene and benzene rings is 80.34 (12)°. The pyrazoline ring is in an envelope conformation and the plane through the four coplanar atoms makes dihedral angles of 85.13 (9)° and 6.89 (10)° with the thiophene and benzene rings, respectively. The acetyl group, except for the hydrogen atoms, is nearly coplanar with the attached pyrazoline ring. An intermolecular C2—H2A···O1(-1 - *x*, 1 - *y*, -*z*) hydrogen bond is found in the crystal structure (Table 1). Further, a short intermolecular Cl4···S21(1-x,1-y,1-z) contact of 3.4250 (13) Å is also found in the crystal structure.

### Experimental

A mixture of 1-(2,4-dichloro-5-fluorophenyl)-3-(3-methylthien-2-yl) prop-2-en-1-one (5 g, 0.016 mol) and a molar equivalent of hydrazine hydrate (80%) in glacial acetic acid (25 ml) was heated on a water bath at 363–365 K for 5–6 h. The reaction mass was then poured into ice-cold water. The solid obtained was filtered, washed with water, dried and crystallized from methanol to yield the title compound. Yield 5.5 g (93.5%).

### Refinement

H atoms were positioned geometrically and allowed to ride on their parent atoms, with C—H = 0.93, 0.96, 0.97 and 0.98 Å for Csp^2^, methyl, methylene and methine C, respectively; *U*_iso_(H) = k*U*_eq_(C), where k = 1.5 for methyl and 1.2 for all other H atoms.

### Crystal data

**Table:**

C_16_H_13_Cl_2_FN_2_OS	*Z* = 2
*M**_r_* = 371.25	*F*(000) = 380
Triclinic, *P*1	*D*_x_ = 1.478 Mg m^−^^3^
Hall symbol: -P 1	Melting point: 369.5 K
*a* = 7.2240 (5) Å	Mo *K*α radiation, λ = 0.71073 Å
*b* = 8.8642 (4) Å	Cell parameters from 4639 reflections
*c* = 14.0518 (9) Å	θ = 4.6–32.4°
α = 100.794 (5)°	µ = 0.53 mm^−^^1^
β = 103.307 (6)°	*T* = 295 K
γ = 101.003 (5)°	Chunk, pale-yellow
*V* = 833.99 (10) Å^3^	0.52 × 0.43 × 0.35 mm

### Data collection

**Table:**

Oxford Diffraction R Gemini diffractometer	5445 independent reflections
Radiation source: fine-focus sealed tube	3028 reflections with *I* > 2σ(*I*)
graphite	*R*_int_ = 0.020
Detector resolution: 10.5081 pixels mm^-1^	θ_max_ = 32.5°, θ_min_ = 4.6°
φ and ω scans	*h* = −10→10
Absorption correction: multi-scan (CrysAlis RED; Oxford Diffraction, 2008)	*k* = −13→13
*T*_min_ = 0.786, *T*_max_ = 1.000	*l* = −21→21
12358 measured reflections	

### Refinement

**Table:**

Refinement on *F*^2^	Primary atom site location: structure-invariant direct methods
Least-squares matrix: full	Secondary atom site location: difference Fourier map
*R*[*F*^2^ > 2σ(*F*^2^)] = 0.051	Hydrogen site location: inferred from neighbouring sites
*wR*(*F*^2^) = 0.179	H-atom parameters constrained
*S* = 1.12	*w* = 1/[σ^2^(*F*_o_^2^) + (0.083*P*)^2^ + 0.1183*P*] where *P* = (*F*_o_^2^ + 2*F*_c_^2^)/3
5445 reflections	(Δ/σ)_max_ = 0.001
210 parameters	Δρ_max_ = 0.32 e Å^−^^3^
0 restraints	Δρ_min_ = −0.39 e Å^−^^3^

### Special details

**Table:**

Geometry. Bond distances, angles *etc*. have been calculated using the rounded fractional coordinates. All su's are estimated from the variances of the (full) variance-covariance matrix. The cell e.s.d.'s are taken into account in the estimation of distances, angles and torsion angles
Refinement. Refinement of *F*^2^ against ALL reflections. The weighted *R*-factor *wR* and goodness of fit *S* are based on *F*^2^, conventional *R*-factors *R* are based on *F*, with *F* set to zero for negative *F*^2^. The threshold expression of *F*^2^ > 2σ(*F*^2^) is used only for calculating *R*-factors(gt) *etc*. and is not relevant to the choice of reflections for refinement. *R*-factors based on *F*^2^ are statistically about twice as large as those based on *F*, and *R*- factors based on ALL data will be even larger.

### Fractional atomic coordinates and isotropic or equivalent isotropic displacement parameters (Å^2^)

**Table:**

	*x*	*y*	*z*	*U*_iso_*/*U*_eq_	
Cl2	0.31301 (12)	0.93549 (8)	0.58594 (5)	0.0696 (3)	
Cl4	0.45313 (12)	0.40815 (13)	0.68787 (6)	0.0875 (4)	
S21	0.33962 (10)	0.82555 (8)	0.17261 (5)	0.0572 (2)	
F5	0.2322 (3)	0.25555 (19)	0.47920 (15)	0.0820 (7)	
O1	−0.2572 (3)	0.7121 (2)	0.06719 (14)	0.0721 (7)	
N1	−0.0630 (3)	0.6973 (2)	0.21112 (13)	0.0432 (5)	
N2	0.0012 (3)	0.6131 (2)	0.28019 (13)	0.0388 (5)	
C1	−0.1967 (4)	0.6281 (3)	0.12037 (17)	0.0499 (7)	
C2	−0.2599 (5)	0.4503 (3)	0.0905 (2)	0.0753 (10)	
C3	0.1042 (3)	0.7120 (2)	0.36402 (15)	0.0352 (6)	
C4	0.1162 (4)	0.8829 (3)	0.36061 (16)	0.0460 (7)	
C5	0.0216 (3)	0.8696 (2)	0.24839 (16)	0.0434 (7)	
C11	0.1899 (3)	0.6464 (3)	0.44812 (15)	0.0383 (6)	
C12	0.2871 (3)	0.7332 (3)	0.54694 (16)	0.0464 (7)	
C13	0.3677 (3)	0.6604 (4)	0.62105 (18)	0.0574 (9)	
C14	0.3507 (4)	0.5009 (4)	0.5990 (2)	0.0574 (9)	
C15	0.2522 (4)	0.4140 (3)	0.5020 (2)	0.0533 (8)	
C16	0.1750 (3)	0.4835 (3)	0.42821 (18)	0.0457 (7)	
C22	0.1646 (3)	0.9275 (3)	0.19146 (16)	0.0429 (6)	
C23	0.1824 (4)	1.0568 (3)	0.15275 (18)	0.0514 (8)	
C24	0.3412 (4)	1.0737 (3)	0.10905 (19)	0.0629 (9)	
C25	0.4390 (4)	0.9588 (4)	0.1136 (2)	0.0634 (10)	
C26	0.0455 (5)	1.1637 (3)	0.1527 (3)	0.0800 (11)	
H2A	−0.39392	0.41733	0.05015	0.1130*	
H2B	−0.24825	0.41020	0.14997	0.1130*	
H2C	−0.17792	0.40987	0.05240	0.1130*	
H4A	0.25124	0.94454	0.38196	0.0552*	
H4B	0.04343	0.93070	0.40277	0.0552*	
H5	−0.08333	0.92575	0.24149	0.0520*	
H13	0.43338	0.72114	0.68588	0.0688*	
H16	0.11130	0.42108	0.36356	0.0549*	
H24	0.37514	1.15693	0.07973	0.0755*	
H25	0.54602	0.95296	0.08813	0.0761*	
H26A	−0.01973	1.15300	0.20427	0.1200*	
H26B	−0.05021	1.13543	0.08824	0.1200*	
H26C	0.11838	1.27147	0.16557	0.1200*	

### Atomic displacement parameters (Å^2^)

**Table:**

	*U*^11^	*U*^22^	*U*^33^	*U*^12^	*U*^13^	*U*^23^
Cl2	0.0784 (5)	0.0659 (4)	0.0470 (4)	0.0100 (3)	0.0044 (3)	−0.0045 (3)
Cl4	0.0679 (5)	0.1559 (8)	0.0819 (5)	0.0599 (5)	0.0340 (4)	0.0827 (5)
S21	0.0645 (4)	0.0652 (4)	0.0544 (4)	0.0279 (3)	0.0223 (3)	0.0251 (3)
F5	0.0976 (14)	0.0656 (10)	0.1059 (14)	0.0393 (10)	0.0337 (11)	0.0495 (10)
O1	0.0727 (13)	0.0737 (12)	0.0585 (11)	0.0147 (10)	−0.0122 (9)	0.0292 (10)
N1	0.0460 (10)	0.0385 (9)	0.0402 (9)	0.0047 (8)	0.0017 (8)	0.0163 (7)
N2	0.0357 (9)	0.0400 (9)	0.0378 (9)	0.0061 (7)	0.0032 (7)	0.0146 (7)
C1	0.0470 (13)	0.0536 (13)	0.0429 (12)	0.0087 (10)	0.0013 (10)	0.0142 (10)
C2	0.082 (2)	0.0553 (15)	0.0583 (16)	0.0023 (14)	−0.0197 (14)	0.0040 (13)
C3	0.0335 (10)	0.0376 (10)	0.0371 (10)	0.0093 (8)	0.0117 (8)	0.0123 (8)
C4	0.0565 (14)	0.0401 (11)	0.0394 (11)	0.0083 (10)	0.0135 (10)	0.0083 (9)
C5	0.0493 (12)	0.0368 (10)	0.0445 (12)	0.0116 (9)	0.0088 (9)	0.0150 (9)
C11	0.0345 (10)	0.0487 (11)	0.0353 (10)	0.0117 (9)	0.0116 (8)	0.0147 (9)
C12	0.0371 (11)	0.0611 (14)	0.0386 (11)	0.0083 (10)	0.0095 (9)	0.0115 (10)
C13	0.0408 (12)	0.095 (2)	0.0397 (12)	0.0185 (13)	0.0095 (10)	0.0242 (13)
C14	0.0424 (12)	0.093 (2)	0.0593 (15)	0.0309 (13)	0.0232 (11)	0.0465 (14)
C15	0.0467 (13)	0.0635 (15)	0.0669 (16)	0.0231 (11)	0.0255 (11)	0.0354 (12)
C16	0.0435 (12)	0.0524 (12)	0.0468 (12)	0.0146 (10)	0.0147 (9)	0.0195 (10)
C22	0.0489 (12)	0.0388 (10)	0.0373 (10)	0.0064 (9)	0.0049 (9)	0.0133 (8)
C23	0.0632 (15)	0.0427 (12)	0.0436 (12)	0.0093 (10)	0.0059 (11)	0.0136 (10)
C24	0.0743 (18)	0.0605 (15)	0.0468 (14)	−0.0042 (13)	0.0134 (12)	0.0209 (12)
C25	0.0626 (17)	0.0799 (19)	0.0507 (14)	0.0115 (14)	0.0215 (12)	0.0215 (13)
C26	0.100 (2)	0.0523 (15)	0.085 (2)	0.0327 (16)	0.0071 (18)	0.0184 (15)

### Geometric parameters (Å, °)

**Table:**

Cl2—C12	1.734 (3)	C14—C15	1.382 (4)
Cl4—C14	1.725 (3)	C15—C16	1.367 (4)
S21—C22	1.728 (2)	C22—C23	1.355 (4)
S21—C25	1.707 (3)	C23—C24	1.417 (4)
F5—C15	1.352 (3)	C23—C26	1.495 (4)
O1—C1	1.219 (3)	C24—C25	1.349 (4)
N1—N2	1.382 (3)	C2—H2A	0.9600
N1—C1	1.360 (3)	C2—H2B	0.9600
N1—C5	1.476 (3)	C2—H2C	0.9600
N2—C3	1.293 (3)	C4—H4A	0.9700
C1—C2	1.503 (4)	C4—H4B	0.9700
C3—C4	1.511 (3)	C5—H5	0.9800
C3—C11	1.475 (3)	C13—H13	0.9300
C4—C5	1.540 (3)	C16—H16	0.9300
C5—C22	1.517 (3)	C24—H24	0.9300
C11—C12	1.399 (3)	C25—H25	0.9300
C11—C16	1.397 (4)	C26—H26A	0.9600
C12—C13	1.399 (4)	C26—H26B	0.9600
C13—C14	1.365 (5)	C26—H26C	0.9600
			
Cl2···C4	3.064 (2)	C11···C15^ii^	3.391 (4)
Cl2···S21^i^	3.6953 (10)	C14···C15^iii^	3.502 (4)
Cl4···N1^ii^	3.488 (2)	C14···C16^iii^	3.514 (4)
Cl4···N2^ii^	3.389 (2)	C15···C14^iii^	3.502 (4)
Cl4···C11^iii^	3.596 (2)	C15···C11^ii^	3.391 (4)
Cl4···C16^iii^	3.524 (3)	C16···Cl4^iii^	3.524 (3)
Cl4···F5	2.917 (2)	C16···C14^iii^	3.514 (4)
Cl4···S21^iii^	3.4250 (13)	C16···C16^ii^	3.600 (3)
Cl4···C1^ii^	3.632 (3)	C22···O1	3.172 (3)
Cl2···H4A	2.8200	C24···O1^vi^	3.408 (3)
Cl2···H4B	2.8400	C5···H26A	2.7500
Cl2···H4A^i^	3.0200	C24···H13^i^	3.0000
Cl2···H4B^iv^	3.0600	C24···H25^x^	3.0400
S21···N1	3.121 (2)	C24···H26B^vi^	3.0700
S21···C3	3.689 (2)	C25···H25^x^	3.1000
S21···Cl2^i^	3.6953 (10)	C26···H5	2.7600
S21···Cl4^iii^	3.4250 (13)	H2A···O1^viii^	2.5800
S21···H4A	3.1800	H2B···N2	2.4200
F5···Cl4	2.917 (2)	H4A···Cl2	2.8200
F5···C4^v^	3.260 (3)	H4A···S21	3.1800
F5···H4B^v^	2.8200	H4A···Cl2^i^	3.0200
O1···C22	3.172 (3)	H4B···Cl2	2.8400
O1···C24^vi^	3.408 (3)	H4B···F5^ix^	2.8200
O1···H5	2.6600	H4B···Cl2^iv^	3.0600
O1···H25^vii^	2.7900	H5···O1	2.6600
O1···H2A^viii^	2.5800	H5···C26	2.7600
O1···H24^vi^	2.6100	H5···H26A	2.1700
N1···S21	3.121 (2)	H13···C24^i^	3.0000
N1···Cl4^ii^	3.488 (2)	H16···N2	2.4000
N2···Cl4^ii^	3.389 (2)	H24···O1^vi^	2.6100
N2···H2B	2.4200	H25···O1^xi^	2.7900
N2···H16	2.4000	H25···C24^x^	3.0400
C1···Cl4^ii^	3.632 (3)	H25···C25^x^	3.1000
C3···S21	3.689 (2)	H26A···C5	2.7500
C4···Cl2	3.064 (2)	H26A···H5	2.1700
C4···F5^ix^	3.260 (3)	H26B···C24^vi^	3.0700
C11···Cl4^iii^	3.596 (2)		
			
C22—S21—C25	91.98 (14)	C22—C23—C26	123.8 (3)
N2—N1—C1	123.08 (19)	C24—C23—C26	124.6 (3)
N2—N1—C5	112.89 (16)	C23—C24—C25	114.3 (3)
C1—N1—C5	123.97 (19)	S21—C25—C24	110.8 (2)
N1—N2—C3	108.80 (17)	C1—C2—H2A	109.00
O1—C1—N1	118.9 (2)	C1—C2—H2B	109.00
O1—C1—C2	123.5 (2)	C1—C2—H2C	109.00
N1—C1—C2	117.6 (2)	H2A—C2—H2B	109.00
N2—C3—C4	113.05 (18)	H2A—C2—H2C	110.00
N2—C3—C11	117.69 (18)	H2B—C2—H2C	109.00
C4—C3—C11	129.25 (19)	C3—C4—H4A	111.00
C3—C4—C5	102.60 (17)	C3—C4—H4B	111.00
N1—C5—C4	101.26 (16)	C5—C4—H4A	111.00
N1—C5—C22	110.98 (18)	C5—C4—H4B	111.00
C4—C5—C22	114.48 (19)	H4A—C4—H4B	109.00
C3—C11—C12	125.8 (2)	N1—C5—H5	110.00
C3—C11—C16	117.75 (19)	C4—C5—H5	110.00
C12—C11—C16	116.5 (2)	C22—C5—H5	110.00
Cl2—C12—C11	122.69 (19)	C12—C13—H13	120.00
Cl2—C12—C13	115.80 (19)	C14—C13—H13	120.00
C11—C12—C13	121.5 (2)	C11—C16—H16	119.00
C12—C13—C14	120.5 (2)	C15—C16—H16	119.00
Cl4—C14—C13	121.7 (2)	C23—C24—H24	123.00
Cl4—C14—C15	120.0 (3)	C25—C24—H24	123.00
C13—C14—C15	118.3 (3)	S21—C25—H25	125.00
F5—C15—C14	119.0 (3)	C24—C25—H25	125.00
F5—C15—C16	119.0 (2)	C23—C26—H26A	110.00
C14—C15—C16	122.0 (3)	C23—C26—H26B	110.00
C11—C16—C15	121.2 (2)	C23—C26—H26C	109.00
S21—C22—C5	119.58 (18)	H26A—C26—H26B	109.00
S21—C22—C23	111.36 (19)	H26A—C26—H26C	109.00
C5—C22—C23	129.0 (2)	H26B—C26—H26C	109.00
C22—C23—C24	111.6 (2)		
			
C25—S21—C22—C5	−177.60 (19)	C4—C5—C22—S21	66.8 (2)
C25—S21—C22—C23	0.6 (2)	C4—C5—C22—C23	−111.1 (3)
C22—S21—C25—C24	−0.2 (2)	C3—C11—C12—Cl2	2.4 (3)
C1—N1—N2—C3	−170.7 (2)	C3—C11—C12—C13	−178.4 (2)
C5—N1—N2—C3	6.6 (3)	C16—C11—C12—Cl2	−178.11 (18)
N2—N1—C1—O1	174.2 (2)	C16—C11—C12—C13	1.1 (3)
C5—N1—C1—O1	−2.8 (4)	C3—C11—C16—C15	179.5 (2)
N2—N1—C1—C2	−6.6 (4)	C12—C11—C16—C15	−0.1 (4)
C5—N1—C1—C2	176.4 (2)	Cl2—C12—C13—C14	178.2 (2)
N2—N1—C5—C4	−11.3 (3)	C11—C12—C13—C14	−1.0 (4)
C1—N1—C5—C4	166.0 (2)	C12—C13—C14—Cl4	178.5 (2)
N2—N1—C5—C22	110.7 (2)	C12—C13—C14—C15	0.0 (4)
C1—N1—C5—C22	−72.1 (3)	Cl4—C14—C15—F5	2.2 (4)
N1—N2—C3—C11	−179.1 (2)	Cl4—C14—C15—C16	−177.6 (2)
N1—N2—C3—C4	1.6 (3)	C13—C14—C15—F5	−179.3 (3)
N2—C3—C4—C5	−8.4 (3)	C13—C14—C15—C16	1.0 (4)
C4—C3—C11—C16	−173.1 (2)	F5—C15—C16—C11	179.3 (2)
N2—C3—C11—C12	−172.7 (2)	C14—C15—C16—C11	−1.0 (4)
N2—C3—C11—C16	7.8 (3)	S21—C22—C23—C24	−0.9 (3)
C11—C3—C4—C5	172.4 (2)	S21—C22—C23—C26	176.7 (2)
C4—C3—C11—C12	6.4 (4)	C5—C22—C23—C24	177.1 (2)
C3—C4—C5—N1	10.9 (2)	C5—C22—C23—C26	−5.3 (4)
C3—C4—C5—C22	−108.6 (2)	C22—C23—C24—C25	0.8 (3)
N1—C5—C22—S21	−47.0 (2)	C26—C23—C24—C25	−176.8 (3)
N1—C5—C22—C23	135.1 (2)	C23—C24—C25—S21	−0.3 (3)

Symmetry codes: (i) −*x*+1, −*y*+2, −*z*+1; (ii) −*x*, −*y*+1, −*z*+1; (iii) −*x*+1, −*y*+1, −*z*+1; (iv) −*x*, −*y*+2, −*z*+1; (v) *x*, *y*−1, *z*; (vi) −*x*, −*y*+2, −*z*; (vii) *x*−1, *y*, *z*; (viii) −*x*−1, −*y*+1, −*z*; (ix) *x*, *y*+1, *z*; (x) −*x*+1, −*y*+2, −*z*; (xi) *x*+1, *y*, *z*.

### Hydrogen-bond geometry (Å, °)

**Table:**

*D*—H···*A*	*D*—H	H···*A*	*D*···*A*	*D*—H···*A*
C2—H2A···O1^viii^	0.96	2.58	3.533 (4)	171

Symmetry codes: (viii) −*x*−1, −*y*+1, −*z*.
